# Cardio-metabolic outcomes in South Asians compared to White Europeans in the United Kingdom: a matched controlled population-based cohort study

**DOI:** 10.1186/s12872-021-02133-z

**Published:** 2021-06-30

**Authors:** Munerah Almulhem, Joht Singh Chandan, Krishna Gokhale, Nicola J. Adderley, Rasiah Thayakaran, Kamlesh Khunti, Abd A. Tahrani, Wasim Hanif, Krishnarajah Nirantharakumar

**Affiliations:** 1 Institute of Applied Health Research, College of Medical and Dental Sciences, University of Birmingham, Birmingham, B152TT UK; 2 Diabetes Research Centre, University of Leicester, Leicester, UK; 3 Centre for Endocrinology, Diabetes and Metabolism, Birmingham Health Partners, Birmingham, UK; 4 Diabetes Department, University Hospitals Birmingham NHS Foundation Trust, Birmingham, UK

**Keywords:** Cardiovascular disease, Hypertension, Type 2 diabetes mellitus, South Asian

## Abstract

**Background:**

There appears to be an inequality in the risk of cardio-metabolic disease between those from a South Asian (SA) background when compared to those of White Europeans (WE) descendance, however, this association has not been explored in a large European cohort. This population-based open retrospective cohort explores the incidence of cardio-metabolic disease in those without pre-existing cardiometabolic disease taken from a large UK primary care database from 1st January 2007 to 31st December 2017.

**Methods:**

A retrospective open cohort matched population-based study using The Health Improvement Network (THIN) database. The outcomes of this study were the incidences of cardio-metabolic events (type 2 diabetes mellitus, hypertension, ischemic heart disease, stroke, heart failure, and atrial fibrillation).

**Results:**

A total of 94,870 SA patients were matched with 189,740 WE patients. SA were at an increased risk of developing: T2DM (adjusted hazard ratio (aHR) 3.1; 95% CI 2.97–3.23); HTN (1.34; 95% CI: 1.29–1.39); ischaemic heart disease (IHD) (1.81; 95% CI: 1.68–1.93) and heart failure (HF) (1.11; 95% CI: 1.003–1.24). However, they were at a lower risk of atrial fibrillation (AF) (0.53; 95% CI: 0.48–0.59) when compared to WE. Of those of SA origin, the Bangladeshi community were at the greatest risk of T2DM, HTN, IHD and HF, but were at the lowest risk of AF in when compared to Indians and Pakistanis.

**Conclusion:**

Considering the high risk of cardio-metabolic diseases in the SA cohort, differential public health measures should be considered in these patients to reduce their risk of disease, which may be furthered tailored depending on their country of origin.

**Supplementary Information:**

The online version contains supplementary material available at 10.1186/s12872-021-02133-z.

## Background

The risk of type 2 diabetes is higher in South Asian (SA) populations resulting in an increased risk of macrovascular and microvascular complications, except for neuropathy and diabetic foot [1, 2]. The UK SA population is diverse with a number of sub-ethnicities, varied cultural-religious practices and lifestyle choices which may have an impact on their risk factors for CVD [3]. Indians are thought to be the most physically active group among SA, while Bangladeshis are the least active [4]. Bangladeshi men have the highest prevalence of smoking, whereas alcohol consumption is lower among Bangladeshi and Pakistani communities [5, 6]. A study in Newcastle reported that Bangladeshis are the most socio-economically disadvantaged group and have the highest CVD risk profile among Indians, Pakistanis and White Europeans (WE) [3]. Nazroo [7] has shown that SAs do not share the same risk of CVD development. One study (including 2867 WEs, 2001 Indians, and 1776 Pakistanis and Bangladeshis participants) identified Indians having a similar prevalence of CVD compared to WE with Pakistanis and Bangladeshis having a higher prevalence. However, these findings could be limited as CVD was self-reported.

Understanding the relationship between ethnicity and cardio-metabolic disease is essential as this could help with screening, prevention and management strategies. Hence, a population-based study was conducted aimed at comparing the cardio-metabolic outcomes between SAs and WEs in the UK. Our secondary aim was to compare cardio-metabolic outcomes in SA subgroups, namely Indians, Pakistanis and Bangladeshis.

## Method

### Study design and data source

A retrospective matched population-based open cohort study was carried out using The Health Improvement Network (THIN), an electronic UK primary care database. The database contains data from 787 primary care practices. THIN is a large database covering approximately 6% of the UK population and is demographically representative of the UK population [8]. The crude prevalence of major chronic conditions and death rates adjusted for demographics and deprivation in THIN are comparable to national estimates [9]. THIN provides longitudinal records with data on sociodemographic characteristics, diagnoses, medical tests, results, prescriptions, and additional information using a hierarchal clinical coding system, called Read Codes [10]. IQVIA provided THIN data access to the University of Birmingham. Use of IQVIA Medical Research Data is approved by the UK Research Ethics. In accordance with this approval, the study protocol was reviewed and approved by an independent scientific review committee (reference number: 18THIN071). IQVIA Medical Research Data incorporates data from The Health Improvement Network (THIN), a Cegedim Database. Reference made to THIN is intended to be descriptive of the data asset licensed by IQVIA. This work used de-identified data provided by patients as a part of their routine primary care. Data extraction was facilitated using the Data Extraction for Epidemiological Research (DExtER) tool [11].

### Population and follow-up period

The study period was 1st January 2007 to 31st December 2017. The year 2007 was chosen as the starting point as the completeness of ethnicity data had greatly improved due to payment incentivised introduced recording of ethnicity in the Quality and Outcomes Framework in the financial year prior [12]. The inclusion criteria were patients: > 35 years old and had recorded ethnicity data.

The exposed cohort consisted of patients who had a GP inputted Read Code for SA ethnicity (self-reported). Those defined as a mixed ethnicity were excluded from the exposed and control cohorts.

Although in this study we examined the risk associated with self-determined ethnicity, future research may also wish to explore the risk stratified by country of birth.

Exposed patients were matched 1:2 by propensity scores to WE controls by age, gender, Townsend deprivation index quintile, and study index year.

The index date was set as the date one year after patient registration with the practice or the date the practice was eligible to contribute. These criteria ensured consistent data quality and adequacy of covariate recording. Practices were eligible to contribute to the study on the later of one year after the date practice started using electronic medical records or one year after the practice was deemed to have been recording data acceptably as evidenced by acceptable mortality recordings [13]. The exit date was set at earliest date among dates of patient transfer from practice, death date, date the practice ceased contribution to the THIN database, outcome event date or study end date.

### Outcomes and covariates

The primary outcome of this study was the incidence of cardio-metabolic events: type 2 diabetes mellitus (T2DM), hypertension (HTN), ischaemic heart disease (IHD), stroke/transient ischaemic attack (TIA), heart failure (HF) and atrial fibrillation (AF). Outcomes were identified by the presence of a Read corresponding to one of these conditions.

Covariates that could impact the development of the outcomes were reported at baseline. These included age at index date, gender, body mass index (BMI), blood pressure, lipid profile, smoking status, and Townsend deprivation quintile. Potential confounders were used as model covariates and were selected on the basis of biological plausibility [14–18].

### Patient and public involvement

Patients and the public were not involved in setting the research question or the outcome measures, nor were they involved in developing plans for the design or implementation of the study. Patients or the public were not asked to advise on interpretation or writing up of results. There are no plans to disseminate the results of the research to study participants, the relevant patient communities or the public.

### Statistical analysis

Baseline characteristics of both SA and WE patients were reported using appropriate descriptive statistics (mean and standard deviation (SD) or median and interquartile range (IQR) for continuous data and proportions for categorical data). Logistic regression was used to calculate crude and adjusted odds ratios (OR and aOR, respectively) for outcomes of interest that were present at baseline. ORs were calculated with 95% confidence intervals (95% CIs) and statistical significance was set at *p* < 0.05. Hazard ratios (HR) and 95% CIs were calculated using Cox regression models for outcomes of interest during the follow-up period. For each outcome, patients with a record of the outcome at baseline were excluded. Adjusted hazard ratios (aHR) for cardio-metabolic outcomes were calculated after adjustment for the baseline covariates listed above. In additional analysis, interactions between ethnicity and age, and ethnicity and sex were examined for each outcome.

Variables were complete except for Townsend score, smoking status, BMI, lipid profile and blood pressure. For Townsend score, BMI, and smoking status, missing indicator categories were used in the adjusted analyses. BMI was treated as a categorical variable and grouped into normal weight (18.5–25 kg/m^2^), overweight (25–30 kg/m^2^) and obese (> 30 kg/m^2^).

A sensitivity analysis was conducted using lower BMI cut points for SA patients as proposed in the literature: normal weight (18.5 to 23 kg/m^2^), overweight (23–27.5 kg/m^2^) and obese (> 27.5 kg/m^2^) for SA [19]. STATA v14.2 was used for statistical analysis.

## Results

### Baseline characteristics

A total of 94,870 SA patients were identified in the dataset and matched to 189,740 WEs. Characteristics of both populations are described in detail in Table 1. Across the entire study the population at baseline was 52.49% were male; median (IQR) age was 41 (35 to 52) years; mean (SD) BMI was 26.6 (5.3) kg/m^2^. Matching parameters of age, gender, and Townsend deprivation quintiles were similar between the groups. Compared to the SA cohort, the WE cohort contained more smokers, had higher levels of total cholesterol and HDL and higher blood pressure, whereas increased overall levels of triglyceride were found in SAs. The SA patients had a higher proportion of patients with IHD, HTN, HF, and T2DM compared to the WE patients at baseline. SAs experienced less AF and stroke/TIA. Median follow-up was 4.3 (IQR 1.7 to 7.4) and 4.2 (IQR 1.7 to 7.4) years, for SAs and WEs, respectively.

**Table 1:** Baseline characteristics of the South Asian and White European populations

Characteristic	South Asian (n = 94,870)	White (n = 189,740)
Male, n (%)	49,795 (52.49)	99,594 (52.49)
Age, years, median (IQR)	41 (35 to 53)	41 (35 to 52)
BMI, mean (SD)	26.2 (4.8)	26.8 (5.6)
BMI category, n (%)		
18.5–25 kg/m^2^	35,963 (37.91)	68,996 (36.36)
25–30 kg/m^2^	31,909 (33.63)	56,424 (29.74)
> 30 kg/m^2^	14,878 (15.68)	37,736 (19.89)
Missing	12,120 (12.78)	26,584 (14.01)
Smoking, n (%)		
Smoker	11,656 (12.29)	52,526 (27.68)
Ex-smoker	8459 (8.92)	40,761 (21.48)
Non-smoker	72,551 (76.47)	91,884 (48.43)
Missing	2204 (2.32)	4569 (2.41)
Townsend, n (%)		
1	10,490 (11.06)	22,496 (11.86)
2	10,682 (11.26)	23,429 (12.35)
3	17,959 (18.93)	35,916 (18.93)
4	20,575 (21.69)	38,745 (20.42)
5	16,069 (16.94)	30,186 (15.91)
Missing	19,095 (20.13)	38,968 (20.54)
Lipid profile		
Total cholesterol (mean (SD))	4.9 (1.05)	5.1 (1.01)
Triglycerides (median (IQR))	1.4 (1 to 1.96)	1.3 (0.9 to 1.89)
HDL (mean (SD))	1.26 (0.35)	1.4 (0.42)
Blood pressure, (mean, (SD))		
Systolic	124.1 (16.15)	126.5 (15.6)
Diastolic	76.7 (9.9)	77.57 (9.8)
Comorbidities, n (%)		
Type 2 diabetes	11,487 (12.11)	8052 (4.24)
Hypertension	14,306 (15.08)	22,670 (11.95)
IHD	4135 (4.36)	5061 (2.6)
Stroke or TIA	1283 (1.35)	2812 (1.48)
Heart failure	562 (0.59)	837 (0.44)
Atrial fibrillation	457 (0.48)	1824 (0.96)
Medication, n (%)		
Lipid-lowering drugs	16,311 (17.19)	20,608 (10.86)

### Ethnicity and prevalent cardio-metabolic disease at baseline

**Fig. 1:**
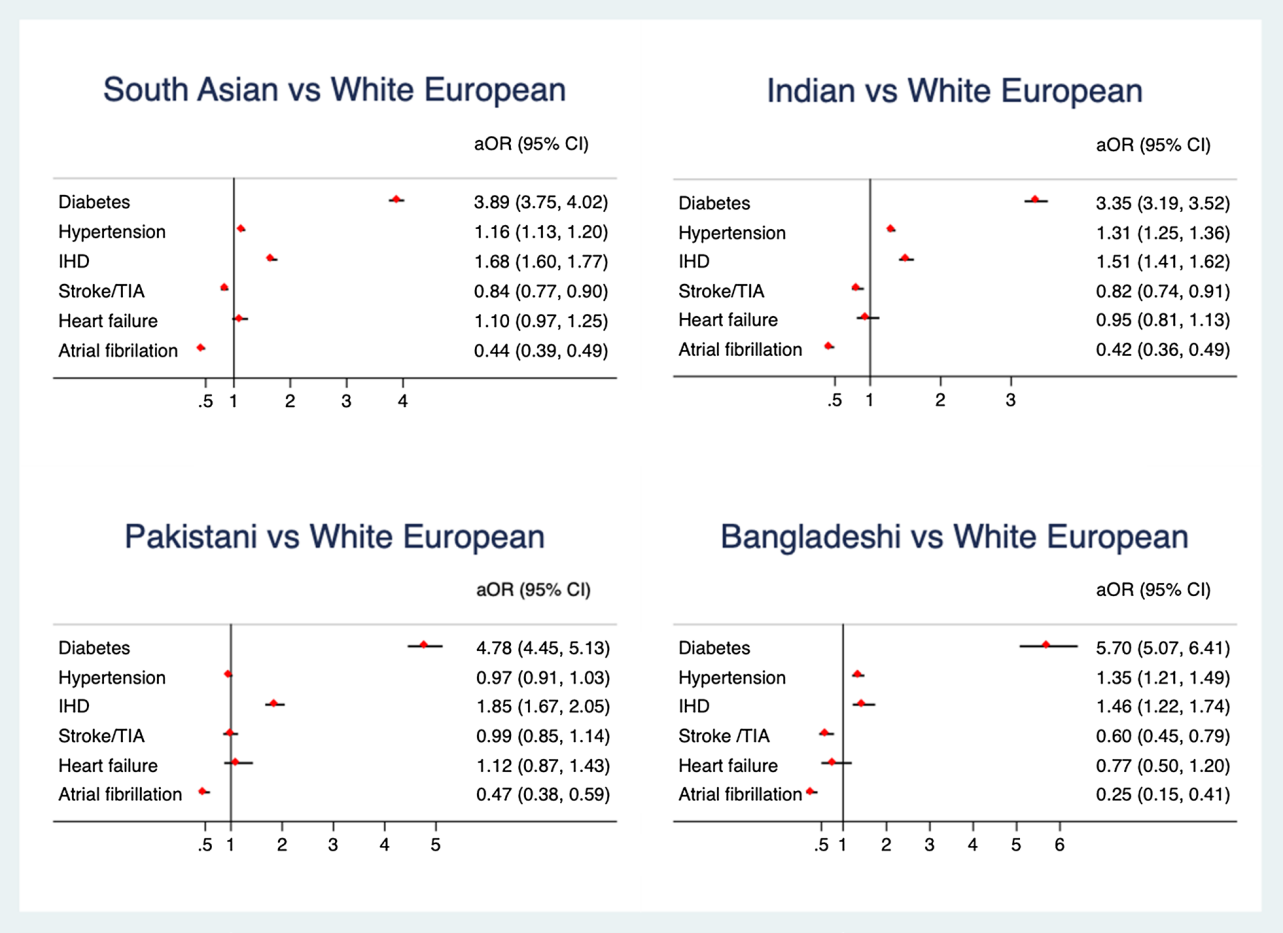
Adjusted odd ratio with 95% confidence intervals for cardio-metabolic risk in south Asians (SA) and the three SA subgroups compared to white Europeans (WE)

Following adjustment, SAs were more likely to have T2DM (aOR 3.89, 95% CI: 3.75–4.02), HTN (aOR 1.16, 95% CI: 1.13–1.20), and IHD (aOR 1.68, 95% CI: 1.60–1.77), compared to WEs at baseline. SAs were less likely to have AF (aOR 0.44, 95% CI: 0.39–0.49) and stroke/ TIA (aOR 0.84, 95% CI: 0.77–0.90) at baseline compared to WEs. The study did not find an association between ethnicity and HF at baseline (aOR 1.1, 95 CI%: 0.97–1.25) (Fig. 1).

### Risk of incident cardio-metabolic disease

**Fig. 2:**
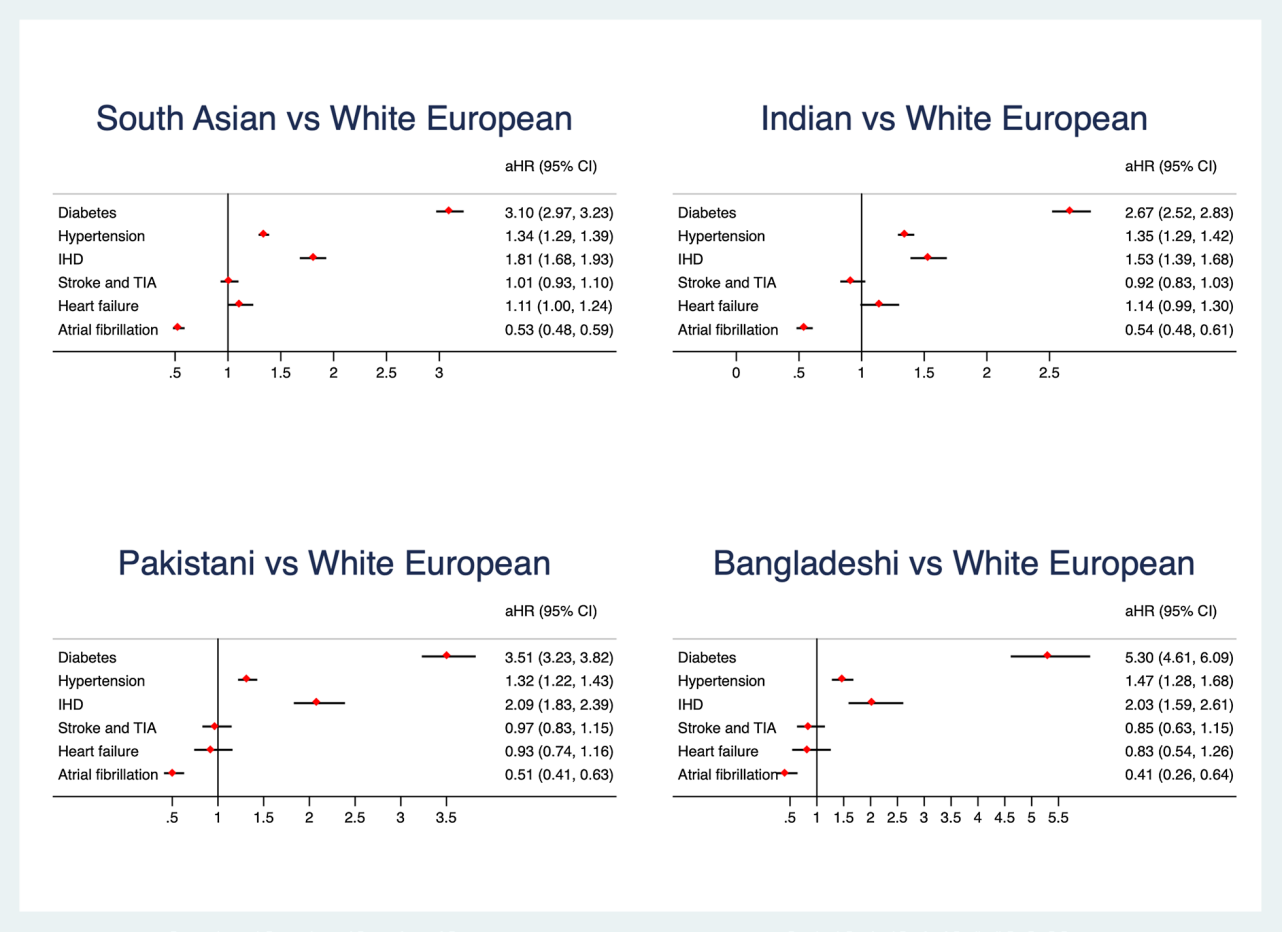
Adjusted Hazard ratios (aHR) with 95% confidence intervals for cardio-metabolic risks in south Asians (SA) and the three SA subgroups compared to white Europeans (WE)

Results are presented in Table 2 and Fig. 2. In the longitudinal analysis, SAs developed 5160 (6.2%) new diagnoses for T2DM compared to WEs who developed 4530 (2.5%) new events. Following adjustment for age, gender, smoking status, BMI category, Townsend deprivation quintile and hypertension, SAs remained at an increased risk of developing T2DM compared to WEs (aHR 3.10; 95% CI: 2.97–3.23, *p* < 0.001).

**Table 2:** Unadjusted and adjusted Hazard ratios (HR) and 95% confidence intervals (95% CI) for White Europeans (WE) and south Asians (SA)

Outcome	Ethnicity	Total n	Incidence n (%)	Person years of follow up	HR (95%CI)	Adjusted HR (95% CI)
T2DM	SA	83,383	5160 (6.19)	370,246	2.54 (2.44–2.65)	3.10 (2.97–3.23)^*^
	WE	181,688	4530 (2.49)	826,498		
HTN	SA	80,564	4998 (6.20)	348,516	1.27 (1.23–1.32)	1.34 (1.29–1.39)^†^
	WE	167,070	8152 (4.88)	724,277		
IHD	SA	90,735	1720 (1.90)	419,025	1.66 (1.56–1.77)	1.81 (1.68–1.93)^‡^
	WE	184,679	2084 (1.13)	848,843		
Stroke/TIA	SA	93,587	988 (1.06)	438,872	0.99 (0.92–1.07)	1.01 (0.93–1.1)^§^
	WE	186,928	1938 (1.04)	863,750		
HF	SA	94,308	642 (0.68)	444,109	1.2 (1.08–1.32)	1.11 (1.003–1.24)^||^
	WE	188,903	1046 (0.55)	876,183		
AF	SA	94,413	615 (0.65)	444,511	0.55 (0.50–0.60)	0.53 (0.48–0.59)^‡^
	WE	184,679	2084 (1.13)	848,843		

^*^Age, gender, smoking, BMI category, Townsend deprivation quintile, hypertension

^†^Age, gender, smoking, BMI category, Townsend deprivation quintile, type 2 diabetes

^‡^Age, gender, smoking, BMI category, Townsend deprivation quintile, type 2 diabetes, hypertension

^§^Age, gender, smoking, BMI category, Townsend deprivation quintile, AF, type 2 diabetes, hypertension

^||^ Age, gender, smoking, BMI category, Townsend deprivation quintile, IHD, type 2 diabetes, hypertension

SAs developed 4998 (6.20%) new diagnoses of HTN compared to 8152 (4.88%) new diagnoses in WEs. After adjusting for age, gender, smoking status, BMI category, Townsend deprivation quintile and T2DM, SAs remained at an increased risk of developing HTN compared to WEs (aHR 1.34; 95% CI: 1.29–1.39, *p* < 0.001).

There were 1720 (1.90%) new IHD diagnoses in SAs compared to 2084 (1.13%) in WEs. After adjustment for age, gender, smoking status, BMI category, Townsend deprivation quintile, hypertension and T2DM, SAs remained at increased risk of developing IHD compared to WEs (aHR 1.81; 95% CI: 1.68–1.93, *p* < 0.001).

Compared to the 988 (1.06%) stroke / TIA events in SAs, there were 1938 (1.04%) stroke /TIA events in WEs. After adjustment for age, gender, smoking status, BMI category, Townsend deprivation quintile, atrial fibrillation, and T2DM, no relationship between ethnicity and stroke /TIA was observed (aHR 1.01; 95% CI: 0.93–1.10, *p* = 0.75).

In the SA cohort, there were 642 (0.68%) new diagnoses of heart failure, compared to 1,046 (0.55%) diagnoses in WEs. Following adjustment age, gender, smoking status, BMI category, Townsend deprivation quintile, HTN, IHD, and T2DM, SAs had an increased risk of developing HF compared to WEs (aHR 1.11; 95% CI: 1.003–1.24, *p* = 0.04).

The longitudinal analysis indicates SAs were at lower risk of developing AF compared to WEs. During the follow up, there were 615 (0.65%) cases and 2084 (1.13%) cases, respectively. After adjusting for age, gender, smoking status, BMI category, Townsend deprivation quintile, HTN and T2DM, SAs remained at lower risk of developing AF compared to their matched WEs (aHR 0.53; 95% CI: 0.48–0.59, *p* < 0.001).

When interaction terms for ethnicity and sex, and ethnicity and age were included in the models, both interactions were found to be statistically significant for the T2DM and HTN outcomes; neither were significant for IHD, stroke/TIA or HF; and the ethnicity and sex interaction was significant for the AF outcome. Introduction of the interaction terms led to an increase in the effect estimate for both T2DM and HTN; there was little impact on the observed aHRs for IHD, stroke/TIA, HF or AF (Additional file 1).

### South Asian subgroup analysis: cardio-metabolic risk

A total of 49,249 Indians, 22,353 Pakistanis, and 7678 Bangladeshi patients were individuals were compared to WE controls in this subgroup analysis. The three cohorts had similar baseline characteristics (Additional files 2–4) when compared to their matched WE controls. The three matched WE cohorts had more smokers, higher levels of total cholesterol and HDL and increased blood pressure compared to the SA groups. Increased levels of triglyceride, except for Indians (similar triglyceride level), were found in SAs.

All three SA subgroups were more likely than their matched WE control to develop T2DM during follow-up (Fig. 2). In particular, the Bangladeshi subgroup had more than a five-fold increased risk of T2DM diabetes during follow-up (aHR 5.30, 95% CI: 4.61–6.09). Indian subgroup had an aHR of 2.67 (95% CI: 2.52–2.83), whereas the Pakistani subgroup had an aHR of 3.51 (95% CI: 3.23–3.82) in comparison to their WE matched control population.

All subgroups had a higher risk of HTN and IHD compared to their respective WE controls. For hypertension, aHR was 1.35 (95% CI: 1.29–1.42) for Indians,

1.32 (95% CI: 1.22–1.43) for Pakistanis and 1.47 (95% CI: 1.28–1.68) for Bangladeshis. For IHD, the aHR was 1.53 (95% CI: 1.39–1.68), 2.09 (95% CI: 1.83–2.39), and 2.03 (95% CI: 1.59–2.61), for Indians, Pakistanis, and Bangladeshis, respectively.

Compared to their matched WE controls, all three SA subgroups had lower risk of AF at follow-up (Bangladeshis: aHR 0.41, 95% CI: 0.26 to 0.64; Indians: aHR 0.54, 95% CI: 0.48–0.61; Pakistanis: aHR: 0.51, 95% CI: 0.41–0.63). There was no significant difference in risk of stroke/TIA or heart failure.

### Sensitivity analysis

**Fig. 3:**
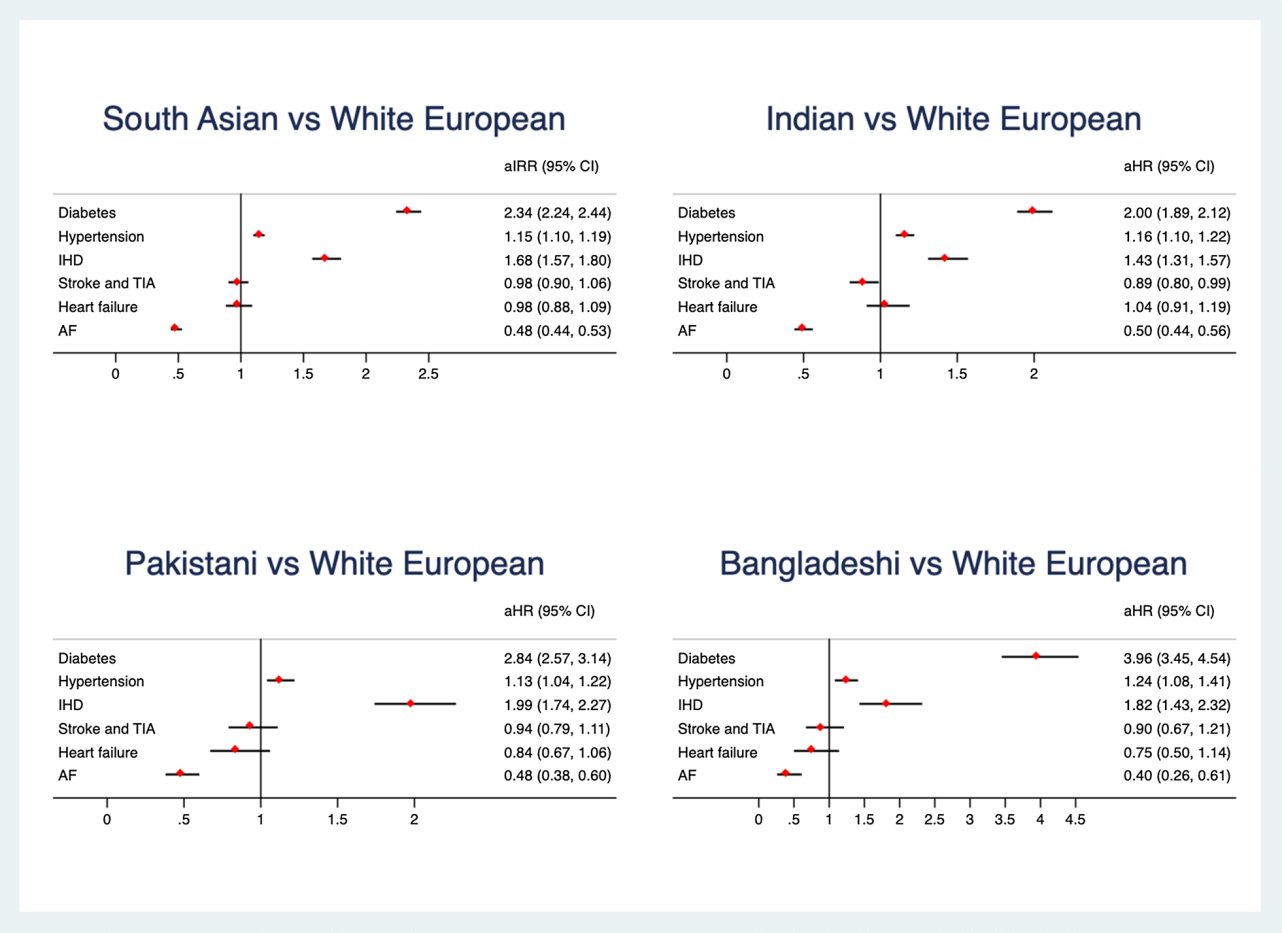
Sensitivity analysis with a different BMI cut point for south Asians. Normal weight 18.5–23 kg/m^2^, overweight 23–27.5 kg/m^2^ and obese > 27.5 kg/m^2^

We conducted a sensitivity analysis assigning different BMI cut off points to SA population as follows: overweight 23–27.5 kg/m^2^ and obese > 27.5 kg/m^2^. Alteration of the BMI cut points decreased the estimated effect size (Fig. 3).

## Discussion

Overall, SA patients were at an increased risk of T2DM, IHD and heart failure, but at a lower risk of AF. When compared to WEs, however, the conventional risk factors such as smoking prevalence, increased cholesterol level and systolic blood pressure were lower in SAs. This suggests that the increased incidence of IHD may result from higher triglycerides level, other lifestyle factors and inherent genetic risks. The cross-sectional analysis of prevalence diagnoses at baseline showed that SAs had a higher occurrence of T2DM, HTN and IHD, but a lower occurrence of stroke/TIA and AF than WEs. It has been suggested that the high prevalence of T2DM diabetes in SAs is the key factor for their increased risk of IHD compared to WEs in the UK [20]. The SA population has consistently been shown to have a lower prevalence of AF despite a high prevalence of AF risk factors [21–23]. WEs are more likely to present factors that increase the risk of stroke, such as AF, smoking, and alcohol consumption [24].Similar to Owusu Adjah, Bellary [25], we did not find significant differences in risk of stroke between SA and WE patients.

Obesity and insulin resistance are the main pathophysiological factors linked to the development of T2DM [26]. A major risk factor for insulin resistance is increased body fat [27] with central obesity an even stronger predictor [28]. Other risk factors associated with insulin resistance include increased blood pressure, hyperglycaemia, central obesity, and dyslipidaemia [29, 30].

It is thought that SAs have greater insulin resistance compared to WEs [31]. Coupled to this, SAs are also known to bear many of the mentioned risk factors for T2DM, namely central obesity and dyslipidaemia, in both prediabetic and diabetic states [26, 32]. Further, SAs are reported to have a higher prevalence of central obesity compared to WEs despite similar or smaller BMIs [33]. In fact, recent evidence suggests that at any age, SAs are at a greater risk of developing T2DM at a lower BMI [34]. This increased risk of T2DM in SAs is posited to stem from inherent impairment of β-cell function rather than insulin resistance [35–37].

Differences in health outcomes across ethnic groups have been documented in the UK [38–40], however, SAs have been tended to be studied as one group. Thus, previous findings may not accurately provide a comprehensive characterization of SA when divided into subgroups. In this study, we have explored variations in outcomes dependent on the SA subgroup. The study indicated that compared to their matched WE controls, Bangladeshis had the highest risk of T2DM. This was consistent with the findings of Hippisley-Cox, Coupland [41] findings, which reported that Bangladeshis had a higher hazard ratio than Pakistanis and Indians when compared to WEs. The heterogeneity across the SA subgroups could be explained by the heterogeneity in health consideration: though the sub groups share the same ethnicity, they bear different risk factors [3]. For example, smoking is known to be more prevalent amongst Bangladeshis men than Indians and Pakistanis [5]. Similar findings were corroborated in our study population. Alcohol consumption is higher in Indians compared to Pakistanis and Bangladeshis [6]. Indians have a lower risk of IHD coupled with the lowest rates of smoking and highest levels of physical activity [42].

The strengths of this study include a large sample size, including separate SA subgroups, which was matched with a white European population. Another strength of this study is the investigation of a wide range of diseases with a large number of events in the different SA groups at baseline and follow-up. Including SA subgroups allowed key insights into the heterogeneities within the greater SA group that are lost when combining all the SA subgroups together. However, a key limitation of this study is that there is a large proportion of patients with missing data for ethnicity, which could affect the generalisability of the study Although, ethnicity information is available for approximately 50% of the primary care population in the total THIN dataset from conception date till present, changes to the Quality Outcomes Framework (incentivised GP payments for improving coding) between 2006 and 2012 improved coding of ethnicity [43]. By 2007 the proportion of patients with a recorded ethnicity improved to 78.3% [43]. Although there were still some patient records with missing ethnicity in the total dataset, to strengthen our approach, we only included records from 2007 onwards. Physical activity level and alcohol consumption are also notable risk factors for cardiovascular disease; however, this information was unavailable at the time of data extraction. Similarly, data on education and diet are not available, therefore we were unable to explore any potential confounding effect of these variables. Moreover, as SAs are considered to be at a higher risk of many cardiometabolic disorders this may lead to a greater predisposition for clinician led investigation of cardiovascular disease in this cohort. Ultimately, this may result in a greater number of diagnoses compared to other ethnic groups and be presented in our results as a possible information bias.

## Conclusion

SA are at higher risk of T2DM and IHD compared to the WEs, but a lower risk of AF. Though SA subgroups share the same ethnicity, they present different risks of certain diseases. Combining different subgroups could over- or underestimate the reality. The findings of this study suggest that there is inequality in health factors across the SA subgroups. Further research with further disaggregated data is needed to explore the difference in outcomes amongst the heterogeneous South Asian population.

## Supplementary Information

Additional file 1: Table 1. Adjusted Hazard ratios (HR) and 95% confidence intervals (95% CI) for White Europeans (WE) and south Asians (SA) using interaction terms.

Additional file 2: Table 2. Baseline characteristics of Indian and White participants.

Additional file 3: Table 3. Baseline characteristics of Pakistani and White participants.

Additional file 4: Table 4. Baseline characteristics of Bangladeshi and white participants.

## Data Availability

The datasets used and/or analysed during the current study are available from the corresponding author on reasonable request.

## Abbreviations

SA: South Asian
WE: White European
HTN: Hypertension
T2DM: Type 2 diabetes mellitus
IHD: Ischaemic heart disease
HF: Heart failure
AF: Atrial fibrillation
THIN: The Health Improvement Network
TIA: Transient ischaemic attack
SD: Standard deviation
IQR: Interquartile range
OR: Odd ratios
aOR: Adjusted odds ratios
HR: Hazard ratios
aHR: Adjusted Hazard ratio
BMI: Body mass index
