# Supplementary material for: Cardio-metabolic outcomes in South Asians compared to White Europeans in the United Kingdom: a matched controlled population-based cohort study
Source: BMC Cardiovasc Disord. 2021 Jun 30;21:320. doi: 10.1186/s12872-021-02133-z (PMC8244230; doi:10.1186/s12872-021-02133-z)
Supplement: Supplementary file 1 — Adjusted Hazard ratios (HR) and 95% confidence intervals (95% CI) for White Europeans (WE) and south Asians (SA) using interaction terms. [file 12872_2021_2133_MOESM1_ESM.docx]

*Supplementary table 1: adjusted Hazard ratios (HR) and 95% confidence intervals (95% CI) for White Europeans (WE) and south Asians (SA) using interaction terms.*

| **Outcome** | **aHR (95% CI)** |
| --- | --- |
| **T2DM** | 5.50 (95%CI:4.67-6.48) |
| **HTN** | 1.63 (95%CI:1.41-1.89) |
| **IHD** | 1.85 (95%CI:1.40-2.45) |
| **Stroke/TIA** | 0.82 (95%CI:0.57-1.19) |
| **HF** | 1.03 (95%CI:0.60-1.177) |
| **AF** | 0.53 (95%CI:0.33-0.86) |
