# Supplementary material for: Cardio-metabolic outcomes in South Asians compared to White Europeans in the United Kingdom: a matched controlled population-based cohort study
Source: BMC Cardiovasc Disord. 2021 Jun 30;21:320. doi: 10.1186/s12872-021-02133-z (PMC8244230; doi:10.1186/s12872-021-02133-z)
Supplement: Supplementary file 2 — Baseline characteristics of Indian and White participants. [file 12872_2021_2133_MOESM2_ESM.docx]

Supplementary Table 2: Baseline characteristics of Indian and White participants

| **Characteristic** | **Indian (n=49,249)** | **White (n= 98,498)** |
| --- | --- | --- |
| **Male, n (%)** | 25,374 (51.52%) | 50,748 (51.52%) |
| **Age, year ,median (IQR)** | 41 (35 to 54) | 41 (35 to 54) |
| **BMI, mean (SD)** | 25.95 (4.6) | 26.97 (5.6) |
| **BMI category**  18.5-25 kg/m2  25-30 kg/m2  >30 kg/m2  Missing | 10,854 (22.04%)  18,824 (38.22%)  13,413 (27.24%)  6,158 (12.50%) | 34,426 (34.95%)  29,487 (29.94%)  20,114 (20.42%)  14,471 (14.69%) |
| **Smoking, n (%)**  Non-smoker  Smoker  Ex-smoker  Missing | 39,351 (79.90%)  4,590 (9.32%)  4,143 (8.41%)  1,164 (2.36%) | 47,860 (48.59%)  26,203 (26.60%)  21,892 (22.23%)  2,543 (2.58%) |
| **Townsend**, n (%)  1  2  3  4  5  Missing | 6,588 (13.38%)  6,039 (12.26%)  10,353 (21.02%)  10,011 (20.33%)  6,011 (12.21%)  10,247 (20.81%) | 13,176 (13.38%)  12,078 (12.26%)  20,706 (21.02%)  20,022 (20.33%)  12,022 (12.21%)  20,494 (20.81%) |
| **Lipid profile**  Total cholesterol (mean (SD))  Triglycerides(median (IQR))  HDL(mean (SD)) | 4.9(1.04)  1.3 (0.94 to 1.90 )  1.3 (0.36) | 5.1 (1.08)  1.32 (0.93 to 1.95)  1.4 (0.42) |
| **Blood pressure, (mean±SD)**  Systolic  Diastolic | 124.67 (16.21)  76.9 (9.86) | 127.08 (15.63)  77.41 (9.72) |
| **Comorbidities, n (%)**  Type 2 diabetes  Hypertension  IHD  Stroke or TIA  Heart failure  Atrial fibrillation | 5,425 (11.02%)  7,925 (16.09%)  2,184 (4.43%)  671 (1.36%)  295 (0.60%)  244 (0.50%) | 4,414 (4.48%)  12,659 (12.85%)  1,628 (1.65%)  3,266 (3.32%)  549 (0.56%)  1,086 (1.10%) |
