# Supplementary material for: Cardio-metabolic outcomes in South Asians compared to White Europeans in the United Kingdom: a matched controlled population-based cohort study
Source: BMC Cardiovasc Disord. 2021 Jun 30;21:320. doi: 10.1186/s12872-021-02133-z (PMC8244230; doi:10.1186/s12872-021-02133-z)
Supplement: Supplementary file 3 — Baseline characteristics of Pakistani and White participants. [file 12872_2021_2133_MOESM3_ESM.docx]

Supplementary Table 3: Baseline characteristics of Pakistani and White participants

| **Characteristic** | **Pakistani (n= 22,353)** | **White (n= 44,706)** |
| --- | --- | --- |
| **Male, n (%)** | 11, 888 (53.18 %) | 23,776 (53.18%) |
| **Age, years, median (IQR)** | 40 (35 to 51) | 40(35 to 51) |
| **BMI, mean (SD)** | 27.17 (5.1) | 26.98 (5.7) |
| **BMI category, n (%)**  18.5-25 kg/m2  25-30 kg/m2  >30 kg/m2  Missing | 3,693 (16.52%)  7,613 (34.06%)  8,219 (36.77%)  2,828 (12.65%) | 15,826 (35.40%)  13,091( 29.28%)  9,245 (20.68%)  6,544 (14.64%) |
| **Smoking, n (%)**  Smoker  Ex-smoker  Non-smoker  Missing | 16,484 (73.74%)  3,473 (15.54%)  1,891 (8.46%)  505 (2.26%) | 20,702 (46.31%)  13,247 (29.63%)  9,581 (21.43%)  1,176 (2.63%) |
| **Townsend, n (%)**  1  2  3  4  5  Missing | 2,005 (8.97%)  2,315 (10.36%)  3,424 (15.32%)  5,275 (23.60%)  4,606 (20.61%)  4,728 (21.15%) | 4,010 (8.97%)  4,630 (10.36%)  6,848 (15.32%)  10,550 (23.60%)  9,212 (20.61%)  9,456 (21.15%) |
| **Lipid profile**  Total cholesterol (mean (SD))  Triglycerides (median (IQR))  HDL (mean (SD)) | 4.8 (1.05)  1.5 (1.01 to 2.1)  1.2 (0.3) | 5.09 (1.1)  1.3(0.91 to 1.99)  1.4 (0.4) |
| **Blood pressure, (mean, (SD))**  Systolic  Diastolic | 123.5 (16.1)  76.4 (9.9) | 126.5 (15.5)  77.3 (9.7) |
| **Comorbidities, n (%)**  Type 2 diabetes  Hypertension  IHD  Stroke or TIA  Heart failure  Atrial fibrillation | 3,145 (14.07%)  153 (0.68%)  1,074 (4.80%)  356 (1.59%)  153 (0.68%)  120 (0.54%) | 1,911 (4.27%)  207 (0.46%)  1,331 (2.98%)  695 (1.55%)  207 (0.46%)  435 (0.97%) |
