# Supplementary material for: Cardio-metabolic outcomes in South Asians compared to White Europeans in the United Kingdom: a matched controlled population-based cohort study
Source: BMC Cardiovasc Disord. 2021 Jun 30;21:320. doi: 10.1186/s12872-021-02133-z (PMC8244230; doi:10.1186/s12872-021-02133-z)
Supplement: Supplementary file 4 — Baseline characteristics of Bangladeshi and white participants. [file 12872_2021_2133_MOESM4_ESM.docx]

Supplementary Table 4: Baseline characteristics of Bangladeshi and white participants

| **Characteristic** | **Bangladeshi (n=7,678)** | **White (n=15,356)** |
| --- | --- | --- |
| **Male, n (%)** | 4,500 (58.61 %) | 9,000 (58.61 %) |
| **Age, years, median (IQR**) | 39(35 to 50) | 39(35 to 50) |
| **BMI, mean (SD)** | 25.77 (4.27) | 27.1 (5.83) |
| **BMI category, n (%)**  18.5-25 kg/m2  25-30 kg/m2  >30 kg/m2  Missing | 1,640 (21.36%)  3,136 (40.84%)  1,982 (25.81%)  920 (11.98%) | 5,251 (34.20%)  4,461 (29.05%)  3,206 (20.88%)  2,438 (15.88%) |
| **Smoking, n (%)**  Smoker  Ex-smoker  Non-smoker  Missing | 4,957 (64.56%)  1,703 (22.18%)  298 (3.88%)  720 (9.38%) | 6,670 (43.44%)  5,109 (33.27%)  1,016 (6.62%)  2,561 (16.68%) |
| **Townsend, n (%)**  1  2  3  4  5  Missing | 393 (5.12%)  605 (7.88%)  994 (12.95%)  1,569 (20.44%)  2,832 (36.88%)  2,570 (16.74%) | 786 (5.12%)  1,210 (7.88%)  1,988 (12.95%)  3,138 (20.44%)  5,664 (36.88%)  1,285 (16.74%) |
| **Lipid profile**  Total cholesterol (mean (SD))  Triglycerides (median (IQR))  HDL (mean (SD)) | 4.8 (1.09)  1.57 (1.1 to 2.20)  1.1 (0.3) | 5.08 (1.10)  1.37 (0.96-2.00)  1.34 (0.4) |
| **Blood pressure, (mean, (SD))**  Systolic  Diastolic | 121.9 (15.71)  76.05 (9.86) | 126.6 (15.6)  77.4 (9.84) |
| **Comorbidities, n (%)**  Type 2 diabetes  Hypertension  IHD  Stroke or TIA  Heart failure  Atrial fibrillation | 1,249 (16.27%)  1,163 (15.15%)  336 (4.38%)  93 (1.21%)  47 (0.61%)  22 (0.29%) | 705 (4.59%)  1,691 (11.01%)  452 (2.94%)  259 (1.69%)  90 (0.59%)  155 (1.01%) |
